# Crystal structure and Hirshfeld surface analysis of 1-[(*E*)-2-(3-nitro­phen­yl)diazen-1-yl]naphthalen-2-ol

**DOI:** 10.1107/S2056989023001068

**Published:** 2023-02-09

**Authors:** Mohamed Amine Benaouida, Ali Benosmane, Mehdi Boutebdja, Hocine Merazig

**Affiliations:** a Department of Chemistry, Faculty of Sciences, University of 20 Août 1955-Skikda, Skikda 21000, Algeria; b Unité de Recherche de Chimie de l’Environnement et Moléculaire Structurale (CHEMS), Faculté des Sciences Exactes, Université Frères Mentouri Constantine 1, Constantine, 25017, Algeria; c Département Tronc Commun Technologie, Université Larbi Ben M’hidi Oum El Bouaghi, Oum El Bouaghi 04000, Algeria; d Laboratoire de Technologie des Matériaux Avancés, École Nationale Polytechnique de Constantine, Nouvelle Ville Universitaire, Ali Mendjeli, Constantine 25000, Algeria; Venezuelan Institute of Scientific Research, Venezuela

**Keywords:** azo dyes, X-ray diffraction, crystal structure, inter­molecular inter­actions, Hirshfeld surface

## Abstract

The title compound, C_16_H_11_N_3_O_3_, belongs to the family of azo dyes. In the light of a single-crystal X-ray study, it is evident that of the tautomeric forms (azo–hydrazone), the hydrazone form is the predominant form in the solid state, namely, (1*E*)-1-[2-(3-nitro­phen­yl)hydrazin-1-yl­idene]-1,2-di­hydro­naphthalen-2-one. The naphthol and benzene fragments attached to the –N=N– moiety adopt the *s-trans* conformation. Furthermore, the mol­ecules are nearly coplanar, subtending a dihedral angle of 2.63 (5)°. An intra­molecular N—H⋯O hydrogen bond occurs. There are only two types of inter­molecular inter­actions in the crystal structure: strong hydrogen-bonding C—H⋯O inter­actions and π–π stacking inter­actions. The importance of C—H⋯O inter­actions in the mol­ecular packing is reflected by the relatively high contributions (28.5%) made by O⋯H/H⋯O contacts to the Hirshfeld surface.

## Abstract 2

The hydrazone form is the predominant form in the solid state. The naphthol and benzene fragments attached to the –N=N– moiety adopt the *s-trans* conformation. There are only two types of inter­molecular inter­actions in the crystal structure: strong hydrogen-bonding C—H⋯O inter­actions and π–π stacking inter­actions.

## 1. Chemical context

Azo compounds, which include the functional group *R*—N=N—*R*′ where *R* and *R*′ can either be aryl or alkyl, aryl azo compounds being more common than aliphatic azo compounds (Christie, 2001 ▸), have striking colors. These colors, particularly reds, oranges, and yellows, are the result of π-electron delocalization through aromatic moieties (Debnath *et al.*, 2015 ▸; Ferreira *et al.*, 2013 ▸). They are therefore used as dyes, not only in textile colorants but in many other industrial fields for coloring different substrates, as printing inks, in biological reactions and in the cosmetics industry (Hunger, 2003 ▸; Ran *et al.*, 2022 ▸; Mathieu-Denoncourt *et al.*, 2014 ▸; Shi & Chen, 2014 ▸; Chudgar & Oakes, 2003 ▸; Benkhaya *et al.*, 2020 ▸).

Detailed knowledge of mol­ecular structures is essential for determining structure–function relationships and for a systematic approach to the design of new dyes. Structural information obtained from single-crystal X-ray diffraction analysis including conformation, stereochemistry, intra- and inter­molecular inter­actions is related to the optical properties of azo dyes (Pavlović *et al.*, 2009 ▸). In the case of 1-phenyl­azo-2-naphthol derivatives, a strong hydrogen bond enhanced by resonance is established, inducing the azo (OH) → hydrazo (NH) tautomeric displacement (Benosmane *et al.*, 2015 ▸; Bougueria, Benaouida *et al.*, 2013 ▸; Bougueria *et al.*, 2014 ▸). This is directly connected with the presence of at least one protic donor group in conjugation to the azo bridge (2-naphthol) (Antonov, 2016 ▸). As a part of our continuing inter­est in the synthesis and crystallography evaluation of azo-2 naphthol compounds, we embarked on the present crystallographic study and report herein the synthesis, mol­ecular structure and Hirshfeld surface analysis of dye derived from 1-phenyl­azo-2-naphtol: (*E*)-1-(3-nitro­phenyl­azo)-2-naphtol. 
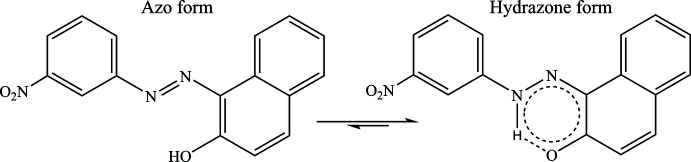


## 2. Structural commentary

**Figure 1:**
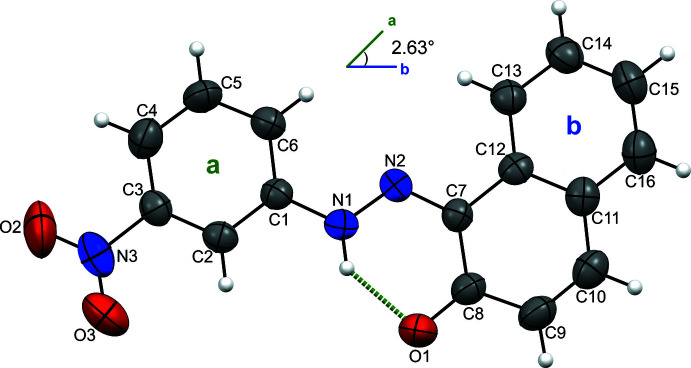
The mol­ecular structure with the atom-labeling scheme. Displacement ellipsoids drawn at the 30% probability level. Intra­molecular hydrogen bonds are shown as dashed lines.

The mol­ecular structure of the title compound (Fig. 1 ▸) was solved in the ortho­rhom­bic space group *P*2_1_2_1_2_1_. The N1—N2, C1—N1, C7—N2 and C8—O1 bond lengths are 1.312 (4), 1.394 (5), 1.330 (5) and 1.276 (5) Å, respectively, indicating that the dye compound has crystallized in the hydrazone tautomeric form (*i.e.* proton transfer from the naphthol group to the azo group); bond lengths and angles are within normal ranges and are comparable to those reported for other azo compounds (Benaouida *et al.*, 2013 ▸; Bougueria, Benosmane *et al.*, 2013 ▸; Mili *et al.*, 2013 ▸; Xu *et al.*, 2010 ▸). The mol­ecule adopts an *s*-trans conformation, with the two aryl groups residing on the opposite side of the azo group. The naphthol and benzene rings attached to the hydrazo group are almost coplanar, subtending a dihedral angle of 2.63 (5)°, indicating significant electron delocalization within the mol­ecule. The mol­ecular structure is stabilized by an intra­molecular N—H⋯O hydrogen bond involving hydrogen atoms from the hydrazo groups (Table 1 ▸).

## 3. Supra­molecular features

**Figure 2:**
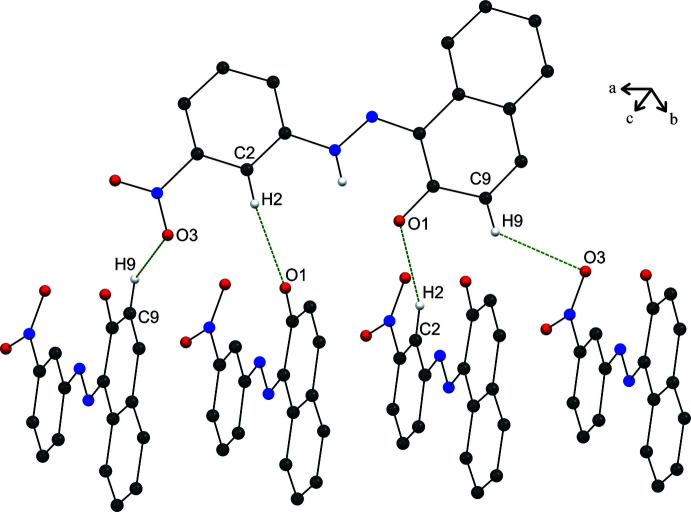
A view along the *c* axis of the crystal packing of the title compound. The C—H⋯O hydrogen bonds are shown as dashed lines (see Table 1 ▸ for details).

**Figure 3:**
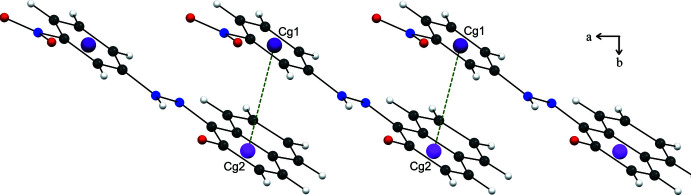
π-π- stacking inter­actions, view along the *c* axis of the stacked mol­ecules. Dashed light-green lines indicate *Cg*1⋯*Cg*2 contacts.

In the crystal, mol­ecules are held together by strong inter­molecular C—H⋯O hydrogen bonds (Table 1 ▸), forming parallel chains propagating along the *a*-axis direction (Fig. 2 ▸). Cohesion of the crystal structure is enhanced by the presence of π–π stacking inter­actions (Fig. 3 ▸), the most significant being between the benzene and naphthalene rings [*Cg*1⋯*Cg*2(1 + *x*, *y*, *z*) = 3.607 (2) Å where *Cg*1 and *Cg*2 are the centroids of the C1–C6 and C7–C12 rings, respectively].

## 4. Database survey

A search for 1-phenyl­azo-2-naphthol derivatives in the Cambridge Structural Database (CSD; Version 2022.3.0, last update November 2022; Groom *et al.*, 2016 ▸), revealed several examples of structurally similar azo-2-naphthol compounds prepared using different aromatic primary amines. The crystal structures of 1-[(*E*)-2-(5-chloro-2 hy­droxy­phen­yl)hydrazin-1-yl­idene]naphthalen-2(1*H*)-one (Bou­gueria *et al.*, 2021 ▸), (4-amino­sulfonyl­phen­yl)[(2-oxidonaphthalen-1-yl)-imino]­aza­­n­ium (Benosmane *et al.*, 2016 ▸), (*E*)-1-(4-fluoro­phen­yl)-2-(2-oxidonaphthalen-1-yl)diazen-1-ium (Bougueria *et al.*, 2017 ▸) have been reported, as well as the structural and optoelectronic properties and theoretical investigation of a novel square-planar nickel (II) complex with an (*o*-tolyl­diazen­yl) naphthalen-2-ol ligand (Benosmane *et al.*, 2023 ▸) that exhibits structural diversity with inter­esting optoelectronic properties.

## 5. Hirshfeld surface analysis

**Figure 4:**
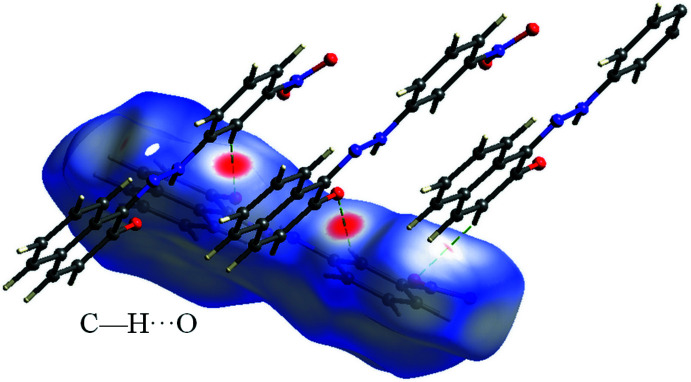
Hirshfeld surfaces mapped over *d*_norm_ in the range −0.23 Å (red) to 1.23 Å (blue).

**Figure 5:**
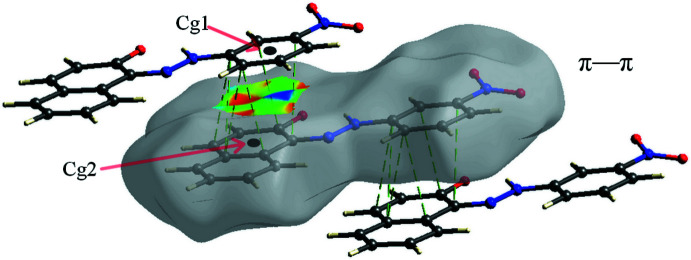
Three-dimensional Hirshfeld surface mapped over shape-index.

**Figure 6:**
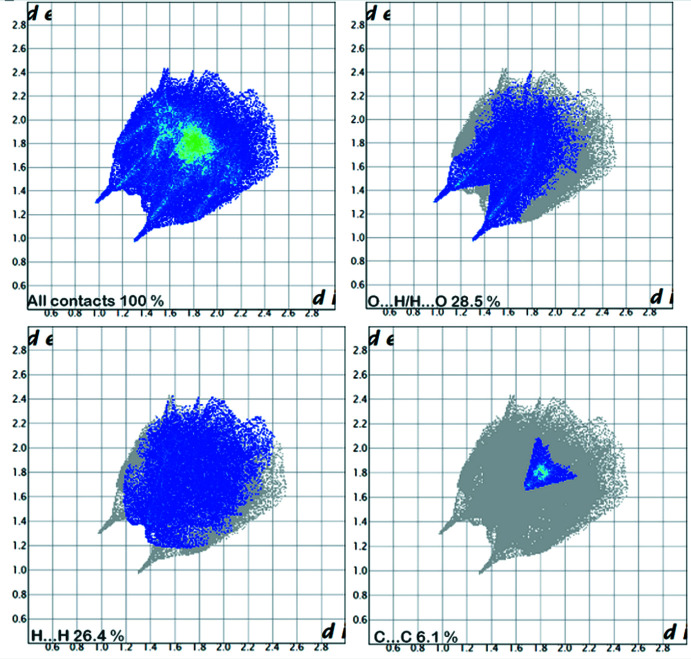
Two-dimensional fingerprint plots.

The supra­molecular inter­actions in the title structure were investigated qu­anti­tatively and visualized with *Crystal Explorer* (Spackman & Jayatilaka, 2009 ▸; McKinnon *et al.*, 2004 ▸). Fig. 4 ▸ shows the Hirshfeld surface mapped over *d*_norm_ in the range −0.2344 (red) a.u. to 1.2354 (blue) a.u. The donors and acceptors of inter­molecular C—H⋯O closest inter­actions in the structure are seen as bright-red spots near the benzene-H2, naphthalene-H9, hydroxyl-O1 and nitro-O3 atoms. The Hirshfeld surface mapped over shape-index is shown in Fig. 5 ▸ where the triangles clearly illustrate the π–π stacking inter­actions. The two-dimensional fingerprint plots are shown in Fig. 6 ▸. H⋯O/O⋯H inter­actions provide the largest contribution (28.5%) to the surface. The second largest contribution is from H⋯H contacts (26.4%). The presence of C⋯C inter­actions (6.1%), corresponding to π–π stacking, is also important. Table 2 ▸ summarizes the percentage contributionsof different types of contacts to the Hirshfeld surface.

## 6. Synthesis and crystallization

The title compound was obtained through the diazo­tization of 3-nitro­aniline followed by a coupling reaction with 2-naphthol. A solution of hydro­chloric acid (12 *M*) and 6 mL of water were added to 3-nitro­aniline (0.02 mol) at 273 K. Sodium nitrite solution (0.02 mol, in 10 mL of water) was added dropwise to the cooled mixture and stirred for 15 min. To the formed diazo­nium salt was added dropwise an aqueous solution of 2-naphthol (0.02 mol in 100 mL of water) containing sodium hydroxide (16 mL). The mixture was then allowed to stir for 1 h at 273 K. The resulting red precipitate was filtered and washed several times with distilled water and dried in air. Red needle-shaped crystals suitable for X-ray analysis were obtained by slow evaporation of an ethanol solution at room temperature (yield 85.4%).

## 7. Refinement details

Crystal data, data collection and structure refinement details are summarized in Table 3 ▸. The hydrogen atom of hydrazo group was localized in a difference-Fourier map and refined with N—H = 0.86 (3) Å with *U*_iso_(H) = 1.2*U*_eq_(N). The other hydrogen atoms were placed in calculated positions with C—H = 0.93 Å and refined using a riding model with fixed isotropic displacement parameters [*U*_iso_(H) = 1.2*U*_eq_(C)].

## Supplementary Material

Crystal structure: contains datablock(s) global, I. DOI: 10.1107/S2056989023001068/zn2025sup1.cif

Supporting information file. DOI: 10.1107/S2056989023001068/zn2025Isup2.cml

CCDC reference: 2239846

Additional supporting information:  crystallographic information; 3D view; checkCIF report

## Figures and Tables

**Table 1:** Hydrogen-bond geometry (Å, °)

*D*—H⋯*A*	*D*—H	H⋯*A*	*D*⋯*A*	*D*—H⋯*A*
N1—H1⋯O1	0.86 (3)	1.84 (3)	2.551 (4)	138 (3)
C2—H2⋯O1^i^	0.93	2.43	3.312 (4)	157
C9—H9⋯O3^ii^	0.93	2.62	3.303 (5)	130

Symmetry codes: (i) 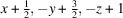 ; (ii) 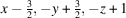 .

**Table 2:** Distribution of individual inter­molecular inter­actions based on Hirshfeld surface analysis

Contact type	Percentage contribution
O⋯H/H⋯O	28.5
H⋯H	26.4
C⋯H/H⋯C	26.0
C⋯C	6.1
N⋯H/H⋯N	4.8
C⋯N/N⋯C	3.8
C⋯O/O⋯C	2.2

**Table 3:** Experimental details

Crystal data	
Chemical formula	C_16_H_11_N_3_O_3_
*M*_r_	293.28
Crystal system, space group	Orthorhombic, *P*2_1_2_1_2_1_
Temperature (K)	296
*a*, *b*, *c* (Å)	6.0981 (9), 14.485 (2), 15.389 (2)
*V* (Å^3^)	1359.3 (3)
*Z*	4
Radiation type	Mo *K*α
μ (mm^−1^)	0.10
Crystal size (mm)	0.50 × 0.30 × 0.10

Data collection	
Diffractometer	Bruker APEXII CCD
No. of measured, independent and observed [*I* > 2σ(*I*)] reflections	12470, 2803, 1342
*R*_int_	0.113
(sin θ/λ)_max_ (Å^−1^)	0.626

Refinement	
*R*[*F* ^2^ > 2σ(*F* ^2^)], *wR*(*F* ^2^), *S*	0.041, 0.087, 0.81
No. of reflections	2803
No. of parameters	203
No. of restraints	1
H-atom treatment	H atoms treated by a mixture of independent and constrained refinement
Δρ_max_, Δρ_min_ (e Å^−3^)	0.12, −0.13
Absolute structure	Flack (1983 ▸), 5026 Friedel pairs
Absolute structure parameter	−2.4 (10)

Computer programs: *APEX2* and *SAINT* (Bruker, 2012 ▸), *SHELXT* (Sheldrick, 2015*a* ▸), *SHELXL2018/3* (Sheldrick, 2015*b* ▸), *Mercury* (Macrae *et al.*, 2020 ▸) and *WinGX* publication routines (Farrugia, 2012 ▸).

## supplementary crystallographic information

### Crystal data

**Table:**

C_16_H_11_N_3_O_3_	*F*(000) = 608
*M**_r_* = 293.28	*D*_x_ = 1.433 Mg m^−^^3^
Orthorhombic, *P*2_1_2_1_2_1_	Mo *K*α radiation, λ = 0.71073 Å
Hall symbol: P 2ac 2ab	Cell parameters from 1342 reflections
*a* = 6.0981 (9) Å	θ = 2.7–18.9°
*b* = 14.485 (2) Å	µ = 0.10 mm^−^^1^
*c* = 15.389 (2) Å	*T* = 296 K
*V* = 1359.3 (3) Å^3^	Needle, red
*Z* = 4	0.50 × 0.30 × 0.10 mm

### Data collection

**Table:**

Bruker APEXII CCD diffractometer	1342 reflections with *I* > 2σ(*I*)
Radiation source: sealed tube	*R*_int_ = 0.113
Graphite monochromator	θ_max_ = 26.4°, θ_min_ = 2.7°
phi and ω scans	*h* = −7→7
12470 measured reflections	*k* = −18→18
2803 independent reflections	*l* = −19→19

### Refinement

**Table:**

Refinement on *F*^2^	Hydrogen site location: inferred from neighbouring sites
Least-squares matrix: full	H atoms treated by a mixture of independent and constrained refinement
*R*[*F*^2^ > 2σ(*F*^2^)] = 0.041	W = 1/[Σ^2^(*F*O^2^) + (0.0223*P*)^2^] WHERE *P* = (*F*O^2^ + 2*F*C^2^)/3
*wR*(*F*^2^) = 0.087	(Δ/σ)_max_ < 0.001
*S* = 0.81	Δρ_max_ = 0.12 e Å^−^^3^
2803 reflections	Δρ_min_ = −0.13 e Å^−^^3^
203 parameters	Extinction correction: shelxl
1 restraint	Absolute structure: Flack (1983), 5026 Friedel pairs
Primary atom site location: structure-invariant direct methods	Absolute structure parameter: −2.4 (10)
Secondary atom site location: difference Fourier map	

### Special details

**Table:**

Geometry. Bond distances, angles etc. have been calculated using the rounded fractional coordinates. All su's are estimated from the variances of the (full) variance-covariance matrix. The cell esds are taken into account in the estimation of distances, angles and torsion angles
Refinement. Refinement on F^2^ for ALL reflections except those flagged by the user for potential systematic errors. Weighted R-factors wR and all goodnesses of fit S are based on F^2^, conventional R-factors R are based on F, with F set to zero for negative F^2^. The observed criterion of F^2^ > 2sigma(F^2^) is used only for calculating -R-factor-obs etc. and is not relevant to the choice of reflections for refinement. R-factors based on F^2^ are statistically about twice as large as those based on F, and R-factors based on ALL data will be even larger.

### Fractional atomic coordinates and isotropic or equivalent isotropic displacement parameters (Å^2^)

**Table:**

	*x*	*y*	*z*	*U*_iso_*/*U*_eq_	
O1	0.1440 (4)	0.7501 (2)	0.56438 (19)	0.0628 (11)	
O2	1.2087 (6)	0.4813 (3)	0.5013 (2)	0.0970 (16)	
O3	0.9538 (5)	0.5490 (2)	0.4279 (2)	0.0808 (11)	
N1	0.3939 (5)	0.6367 (2)	0.6436 (2)	0.0463 (12)	
N2	0.2663 (5)	0.6417 (2)	0.7121 (2)	0.0428 (11)	
N3	1.0269 (7)	0.5166 (3)	0.4945 (3)	0.0623 (17)	
C1	0.5740 (6)	0.5775 (3)	0.6452 (2)	0.0403 (12)	
C2	0.7054 (6)	0.5749 (2)	0.5720 (2)	0.0437 (12)	
C3	0.8870 (6)	0.5181 (3)	0.5725 (3)	0.0457 (12)	
C4	0.9420 (6)	0.4646 (3)	0.6429 (3)	0.0597 (17)	
C5	0.8071 (7)	0.4677 (3)	0.7151 (3)	0.0603 (17)	
C6	0.6243 (6)	0.5242 (3)	0.7173 (3)	0.0490 (14)	
C7	0.0931 (6)	0.6974 (3)	0.7091 (3)	0.0397 (12)	
C8	0.0303 (6)	0.7519 (3)	0.6340 (3)	0.0480 (16)	
C9	−0.1635 (6)	0.8064 (3)	0.6396 (3)	0.0583 (17)	
C10	−0.2879 (7)	0.8059 (3)	0.7116 (3)	0.0567 (16)	
C11	−0.2361 (6)	0.7522 (3)	0.7869 (3)	0.0470 (14)	
C12	−0.0426 (6)	0.6988 (3)	0.7871 (3)	0.0417 (12)	
C13	0.0082 (6)	0.6472 (3)	0.8611 (3)	0.0503 (16)	
C14	−0.1283 (7)	0.6474 (3)	0.9321 (3)	0.0580 (17)	
C15	−0.3181 (7)	0.6989 (3)	0.9312 (3)	0.0627 (17)	
C16	−0.3733 (7)	0.7510 (3)	0.8599 (3)	0.0623 (17)	
H1	0.357 (6)	0.667 (2)	0.5978 (18)	0.063 (15)*	
H2	0.67210	0.61060	0.52350	0.0530*	
H4	1.06630	0.42750	0.64210	0.0720*	
H5	0.83990	0.43110	0.76300	0.0720*	
H6	0.53620	0.52640	0.76660	0.0590*	
H9	−0.20470	0.84290	0.59260	0.0700*	
H10	−0.41370	0.84220	0.71260	0.0680*	
H13	0.13610	0.61220	0.86230	0.0600*	
H14	−0.09250	0.61260	0.98090	0.0700*	
H15	−0.40990	0.69840	0.97940	0.0750*	
H16	−0.50180	0.78550	0.86010	0.0750*	

### Atomic displacement parameters (Å^2^)

**Table:**

	*U*^11^	*U*^22^	*U*^33^	*U*^12^	*U*^13^	*U*^23^
O1	0.0707 (19)	0.0686 (19)	0.0491 (18)	0.0118 (17)	0.0061 (17)	0.0121 (18)
O2	0.059 (2)	0.125 (3)	0.107 (3)	0.023 (2)	0.021 (2)	−0.022 (2)
O3	0.093 (2)	0.095 (2)	0.0545 (19)	−0.014 (2)	0.019 (2)	−0.002 (2)
N1	0.047 (2)	0.054 (2)	0.038 (2)	0.0038 (18)	−0.0002 (19)	0.0050 (19)
N2	0.0433 (19)	0.0452 (19)	0.0399 (18)	0.0002 (17)	0.0032 (17)	−0.0004 (17)
N3	0.062 (3)	0.065 (3)	0.060 (3)	−0.012 (2)	0.016 (3)	−0.018 (2)
C1	0.039 (2)	0.043 (2)	0.039 (2)	−0.0021 (19)	−0.002 (2)	0.000 (2)
C2	0.047 (2)	0.042 (2)	0.042 (2)	−0.006 (2)	0.000 (2)	0.002 (2)
C3	0.046 (2)	0.046 (2)	0.045 (2)	−0.004 (2)	0.004 (2)	−0.006 (2)
C4	0.052 (3)	0.053 (3)	0.074 (3)	0.006 (2)	0.003 (3)	0.000 (3)
C5	0.064 (3)	0.054 (3)	0.063 (3)	0.009 (2)	0.002 (3)	0.019 (3)
C6	0.051 (2)	0.053 (3)	0.043 (2)	0.000 (2)	0.003 (2)	0.005 (2)
C7	0.041 (2)	0.037 (2)	0.041 (2)	0.000 (2)	−0.001 (2)	−0.004 (2)
C8	0.051 (3)	0.044 (2)	0.049 (3)	0.005 (2)	−0.004 (2)	0.004 (2)
C9	0.064 (3)	0.049 (3)	0.062 (3)	0.011 (2)	−0.006 (3)	0.008 (2)
C10	0.057 (3)	0.047 (2)	0.066 (3)	0.007 (2)	−0.005 (3)	−0.004 (3)
C11	0.043 (2)	0.043 (2)	0.055 (3)	−0.004 (2)	−0.001 (2)	−0.009 (3)
C12	0.042 (2)	0.042 (2)	0.041 (2)	−0.003 (2)	−0.008 (2)	−0.004 (2)
C13	0.050 (3)	0.055 (3)	0.046 (2)	0.000 (2)	−0.002 (2)	−0.003 (2)
C14	0.062 (3)	0.067 (3)	0.045 (3)	−0.001 (3)	0.003 (2)	−0.002 (3)
C15	0.058 (3)	0.071 (3)	0.059 (3)	0.000 (3)	0.015 (3)	−0.010 (3)
C16	0.057 (3)	0.058 (3)	0.072 (3)	0.005 (3)	0.010 (3)	−0.010 (3)

### Geometric parameters (Å, º)

**Table:**

O1—C8	1.276 (5)	C10—C11	1.431 (6)
O2—N3	1.225 (6)	C11—C16	1.401 (6)
O3—N3	1.212 (5)	C11—C12	1.411 (5)
N1—N2	1.312 (4)	C12—C13	1.397 (6)
N1—C1	1.394 (5)	C13—C14	1.374 (6)
N2—C7	1.330 (5)	C14—C15	1.377 (6)
N3—C3	1.473 (6)	C15—C16	1.374 (6)
C1—C2	1.383 (5)	C2—H2	0.9300
C1—C6	1.386 (6)	C4—H4	0.9300
N1—H1	0.86 (3)	C5—H5	0.9300
C2—C3	1.380 (5)	C6—H6	0.9300
C3—C4	1.374 (6)	C9—H9	0.9300
C4—C5	1.383 (6)	C10—H10	0.9300
C5—C6	1.383 (6)	C13—H13	0.9300
C7—C8	1.451 (6)	C14—H14	0.9300
C7—C12	1.458 (6)	C15—H15	0.9300
C8—C9	1.424 (5)	C16—H16	0.9300
C9—C10	1.343 (6)		
			
N2—N1—C1	119.2 (3)	C11—C12—C13	118.7 (4)
N1—N2—C7	118.5 (3)	C7—C12—C11	118.7 (4)
O2—N3—O3	124.5 (4)	C7—C12—C13	122.5 (4)
O2—N3—C3	117.4 (4)	C12—C13—C14	120.9 (4)
O3—N3—C3	118.1 (4)	C13—C14—C15	120.2 (4)
N1—C1—C2	117.3 (3)	C14—C15—C16	120.8 (4)
N1—C1—C6	122.1 (3)	C11—C16—C15	120.1 (4)
C2—C1—C6	120.6 (4)	C1—C2—H2	121.00
N2—N1—H1	118 (2)	C3—C2—H2	121.00
C1—N1—H1	122 (2)	C3—C4—H4	121.00
C1—C2—C3	118.5 (3)	C5—C4—H4	121.00
N3—C3—C4	119.5 (4)	C4—C5—H5	119.00
N3—C3—C2	118.0 (4)	C6—C5—H5	119.00
C2—C3—C4	122.5 (4)	C1—C6—H6	120.00
C3—C4—C5	118.0 (4)	C5—C6—H6	120.00
C4—C5—C6	121.2 (4)	C8—C9—H9	119.00
C1—C6—C5	119.2 (4)	C10—C9—H9	119.00
C8—C7—C12	119.9 (3)	C9—C10—H10	118.00
N2—C7—C8	124.6 (4)	C11—C10—H10	118.00
N2—C7—C12	115.5 (4)	C12—C13—H13	120.00
C7—C8—C9	118.2 (4)	C14—C13—H13	120.00
O1—C8—C9	120.9 (4)	C13—C14—H14	120.00
O1—C8—C7	120.9 (3)	C15—C14—H14	120.00
C8—C9—C10	121.1 (4)	C14—C15—H15	120.00
C9—C10—C11	123.1 (4)	C16—C15—H15	120.00
C12—C11—C16	119.4 (4)	C11—C16—H16	120.00
C10—C11—C12	119.0 (4)	C15—C16—H16	120.00
C10—C11—C16	121.6 (4)		
			
C1—N1—N2—C7	−179.0 (3)	C12—C7—C8—C9	0.7 (6)
N2—N1—C1—C2	−179.4 (3)	N2—C7—C12—C11	−176.4 (4)
N2—N1—C1—C6	−0.7 (5)	N2—C7—C12—C13	1.7 (6)
N1—N2—C7—C8	1.8 (6)	C8—C7—C12—C11	1.3 (6)
N1—N2—C7—C12	179.4 (3)	C8—C7—C12—C13	179.4 (4)
O2—N3—C3—C2	165.7 (4)	O1—C8—C9—C10	177.6 (4)
O2—N3—C3—C4	−13.5 (6)	C7—C8—C9—C10	−1.5 (6)
O3—N3—C3—C2	−15.4 (6)	C8—C9—C10—C11	0.4 (7)
O3—N3—C3—C4	165.5 (4)	C9—C10—C11—C12	1.7 (7)
N1—C1—C2—C3	178.9 (3)	C9—C10—C11—C16	−177.9 (4)
C6—C1—C2—C3	0.0 (6)	C10—C11—C12—C7	−2.5 (6)
N1—C1—C6—C5	−179.2 (4)	C10—C11—C12—C13	179.4 (4)
C2—C1—C6—C5	−0.4 (6)	C16—C11—C12—C7	177.2 (4)
C1—C2—C3—N3	−179.4 (4)	C16—C11—C12—C13	−1.0 (6)
C1—C2—C3—C4	−0.3 (6)	C10—C11—C16—C15	−179.8 (4)
N3—C3—C4—C5	180.0 (4)	C12—C11—C16—C15	0.6 (6)
C2—C3—C4—C5	0.8 (6)	C7—C12—C13—C14	−177.4 (4)
C3—C4—C5—C6	−1.2 (6)	C11—C12—C13—C14	0.7 (6)
C4—C5—C6—C1	1.0 (6)	C12—C13—C14—C15	−0.1 (7)
N2—C7—C8—O1	−0.9 (6)	C13—C14—C15—C16	−0.3 (7)
N2—C7—C8—C9	178.2 (4)	C14—C15—C16—C11	0.0 (7)
C12—C7—C8—O1	−178.4 (4)		

### Hydrogen-bond geometry (Å, º)

**Table:**

*D*—H···*A*	*D*—H	H···*A*	*D*···*A*	*D*—H···*A*
N1—H1···O1	0.86 (3)	1.84 (3)	2.551 (4)	138 (3)
C2—H2···O1^i^	0.93	2.43	3.312 (4)	157
C9—H9···O3^ii^	0.93	2.62	3.303 (5)	130

Symmetry codes: (i) *x*+1/2, −*y*+3/2, −*z*+1; (ii) *x*−3/2, −*y*+3/2, −*z*+1.
